# The Capsid Protein of Hepatitis E Virus Inhibits Interferon Induction via Its N-Terminal Arginine-Rich Motif

**DOI:** 10.3390/v11111050

**Published:** 2019-11-11

**Authors:** Shaoli Lin, Yonglin Yang, Yuchen Nan, Zexu Ma, Liping Yang, Yan-Jin Zhang

**Affiliations:** 1 Molecular Virology Laboratory, VA-MD College of Veterinary Medicine and Maryland Pathogen Research Institute, University of Maryland, College Park, MD 20740, USA; lsl1990@umd.edu (S.L.); easing@163.com (Y.Y.); nanyuchen2015@nwsuaf.edu.cn (Y.N.); mazexu@gmail.com (Z.M.); liping.yang@nih.gov (L.Y.); 2 Department of General Practice, Nanjing Medical University, Nanjing 210006, China; 3 College of Veterinary Medicine, Northeast A&F University, Yangling 712100, China

**Keywords:** hepatitis E virus (HEV), interferon (IFN), the capsid protein, arginine-rich motif, IRF3 phosphorylation

## Abstract

Hepatitis E virus (HEV) causes predominantly acute and self-limiting hepatitis. However, in HEV-infected pregnant women, the case fatality rate because of fulminant hepatitis can be up to 30%. HEV infection is zoonotic for some genotypes. The HEV genome contains three open reading frames: ORF1 encodes the non-structural polyprotein involved in viral RNA replication; ORF2 encodes the capsid protein; ORF3 encodes a small multifunctional protein. Interferons (IFNs) play a significant role in the early stage of the host antiviral response. In this study, we discovered that the capsid protein antagonizes IFN induction. Mechanistically, the capsid protein blocked the phosphorylation of IFN regulatory factor 3 (IRF3) via interaction with the multiprotein complex consisting of mitochondrial antiviral-signaling protein (MAVS), TANK-binding kinase 1 (TBK1), and IRF3. The N-terminal domain of the capsid protein was found to be responsible for the inhibition of IRF3 activation. Further study showed that the arginine-rich-motif in the N-terminal domain is indispensable for the inhibition as mutations of any of the arginine residues abolished the blockage of IRF3 phosphorylation. These results provide further insight into HEV interference with the host innate immunity.

## 1. Introduction 

Hepatitis E virus (HEV) is the causative agent of liver inflammation that mainly presents as acute and self-limiting hepatitis [1]. However, in HEV-infected pregnant women, liver inflammation can be exacerbated to fulminant hepatitis and results in up to 30% case fatality [2]. Chronic HEV infection with rapid progression in immunocompromised patients has been a challenge in many countries in recent years [3]. The transmission of HEV is mainly through contaminated drinking water or foods [1]. HEV is a single-stranded, positive-sense RNA virus of the family *Hepeviridae* [4]. HEV strains are classified into two genera: *Orthohepevirus* and *Piscihepevirus*. The genus *Orthohepevirus* contains the previously known genotype 1-4 and the newly recognized genotype 5-8. The genotype 1 and 2 are restricted to humans; genotype 3 and 4 cause zoonotic infections; genotype 5 and 6 are only reported to infect wild boars; genotype 7 and 8 are isolated from camels, while a sole case of human infection from genotype 7 was reported. Among the genotypes that infect humans, genotype 1 is mainly distributed in Asia and Africa; genotype 2 is discovered in Mexico and Africa; genotype 3 is prevalent in industrialized countries; and genotype 4 is discovered in China initially and later isolated in other countries [1]. 

The complete genome of HEV is approximately 7.2 kb with a 5′ cap and 3′ poly(A) [4]. HEV genome encodes three open-reading frames (ORFs): ORF1, ORF2, and ORF3. An additional ORF, ORF4, embedded in ORF1, is reported for genotype 1 strains only and expressed under stress conditions [5]. ORF1 is the largest ORF in the HEV genome and encodes a polyprotein that consists of eight putative functional domains, namely methyltransferase domain (Met), Y domain (Y), papain-like cysteine protease (PCP), hypervariable region (HVR), proline-rich region (Pro), X domain, helicase domain (Hel), and RNA-dependent RNA polymerase domain (RdRp) [6]. Both ORF2 and ORF3 are translated from the sub-genomic RNA of 2.2 kb in alternative frames [7]. ORF2 encodes the capsid protein of 660 amino acids (aa), the major component of HEV virions, and the most immunogenic HEV protein [8]. ORF3, which is partially overlapped with ORF2, is the smallest ORF encoding a protein of 13 kDa. The ORF3 product is a transmembrane protein that resembles class I viroporins [9]. The ion channel activity of the ORF3 product is important in the process of virus release [9]. Among the products of the three ORFs of HEV, only ORF2 product can be visualized in HEV human liver samples or HEV-infected cells by immunohistochemistry (IHC), whereas ORF1 expression is too low to be detectable by IHC in formalin-fixed paraffin-embedded (FFPE) liver samples [10,11]. For ORF3 product, it can be visualized in liver sections of only genotype 1 but not genotype 3 HEV-infected human liver chimeric mice by immunofluorescence assay (IFA) [10]. In HEV-infected HepG2/C3A cells, the level of genomic RNA encoding ORF1 product is also much lower than the subgenomic RNA encoding ORF2/3 at 5 dpi [12]. 

Cytoplasmic viral RNA is recognized by host retinoic acid-inducible gene I (RIG-I)-like receptors (RLRs), including RIG-I and melanoma differentiation-associated gene 5 (MDA5) [13]. The RNA interaction leads to the activation of RLR receptors, which convert mitochondrial antiviral signaling protein (MAVS) into prion-like polymers [14]. The MAVS then binds several E3 ligases, including TNF receptor-associated factor 2, 3, and 6 (TRAF2, 3, and 6) [15], followed by the recruitment and activation of serine/threonine-protein kinase TANK-binding kinase 1 (TBK1). The activated TBK1 then phosphorylates MAVS, leading to the recruitment of interferon regulatory factor 3 (IRF3) to MAVS and subsequent phosphorylation of IRF3 by TBK1 [16]. Upon phosphorylation, IRF3 is homodimerized, dissociated from MAVS, and translocated into the nucleus to activate the expression of type I and III IFNs.

In human hepatoma cells and primary human hepatocytes, HEV infection induces barely detectable type I IFNs though RIG-I and MDA5 expression is elevated [12]. HEV is known to antagonize the IFN production via X domain-mediated blocking of the phosphorylation of IRF3 and the PCP-induced deubiquitination of RIG-I and TBK-1 [17]. The HepG2 cells harboring an HEV subgenomic replicon also have RIG-I signaling impaired [12]. In contrast, the ORF3 product can extend the RIG-I half-life and enhance the activation of RIG-I signaling in HeLa cells [18]. However, ORF2 encodes the most abundant protein in HEV infection. The role of ORF2 product in antagonizing the IFN signaling is not known. 

In this study, we demonstrated that the capsid protein of both genotype 1 and 3 HEV could inhibit poly(I:C)-induced IFN production via blocking IRF3 phosphorylation. The capsid protein could interact with the MAVS-TBK1-IRF3 complex. The N-terminal 111 residues of the capsid protein were shown to be essential to the inhibition of IFN induction. Furthermore, the arginine-rich-motif (ARM) within the N terminus of the capsid protein is indispensable for the IFN inhibition. This study provides further insight into the HEV interference of host innate immunity.

## 2. Materials and Methods

### 2.1. Cells and Viruses

HepG2/C3A (ATCC CRL-10741), HEK293 (ATCC^®^ CRL-1573^™^), HEK293T (ATCC^®^ CRL-3216^™^) and HeLa (ATCC^®^ CCL-2^™^) cells were maintained in Dulbecco’s modified Eagle’s medium (DMEM) supplemented with 10% fetal bovine serum (FBS). HEK293 cells stably expressing IRF3 (HEK293-IRF3) were established previously [17]. The HEV genotype 3 Kernow-C1 strain p6 [19] was used to infect HepG2/C3A cells at a multiplicity of infection (MOI) of 1. The infected HepG2/C3A cells were passaged five times to produce stably infected cells. Sendai virus (ATCC VR-907) was used to induce IFN synthesis.

### 2.2. Plasmids

ORF2 of genotype 1 and 3 HEV were amplified by PCR with the template of Sar55 infectious clone (GenBank Accession Number: AF444002) and Kernow-C1 p6 (GenBank Accession Number: JQ679013), respectively, and were then cloned into the vector pCAGEN [20] (a gift from Connie Cepko (Addgene plasmid #11160; http://n2t.net/addgene:11160; RRID:Addgene_11160)) with a hemagglutinin (HA) tag at the N terminus. The Kernow ORF2 was also cloned into pCAGEN with HA tag at the C terminus. HEV ORF2 truncations D1, D5, and D6 were cloned into pCAGEN vector with the HA tag at the C terminus, and D2, D3, and D4 were cloned into pCAGEN vector with the HA tag at the N terminus. ORF2 truncations D7, D8, D9, and D10 were cloned into a pCDNA3-VenusN1 vector. Mutant pORF2-D7M1 (R28A and R29A), pORF2-D7M2 (R30A and R32A), and pORF2-D7M3 (R32A and R33A) were constructed in a pCDNA3-VenusN1 vector. Mutant full-length ORF2-M1 (R28A and R33A) was cloned into pCAGEN vector with HA tag at the C terminus. VenusC1-IRF3 was previously described [17]. Mutant VenusC1-IRF3 (S385A/S386A) was inserted into pCDNA3-VenusC1 vector. Primers used in this study are listed in Table 1. All in-house-constructed plasmids were subjected to DNA sequencing. The construction of Myc-MAVS and FLAG-TBK1 [21] plasmids was described previously.

### 2.3. Transfection

Transfection of HEK293T and HEK293-IRF3 with plasmid DNA was performed by using FuGene HD (Promega, Madison, WI) according to the instructions of the manufacturer. Transfection of HepG2/C3A was done with Lipofectamine 3000 Reagent (Thermo Fisher Scientific, Waltham, MA). Low molecular weight Poly(I:C), a synthetic analog of dsRNA (Invivogen, San Diego, CA), was used as an inducer for interferon production. The HEK293T and HepG2/C3A cells were transfected with poly(I:C) at a concentration of 1 µg/mL and 6 µg/mL, respectively, followed by incubation at 37 °C before harvesting for further analysis.

### 2.4. Reporter Assay and Cell Viability Assay

HEK293T cells were transfected with the ORF2 plasmids, reporter pGL3.0-IFN-β promoter plasmid, and the Renilla vector pRL-TK (Promega). One day after the transfection, the cells were then transfected with poly(I:C) at the final concentration of 1 µg/mL for another day, followed by cell lysis for firefly and Renilla luciferase activity assay by following the manufacturer’s instructions (Promega). Lysate of the cells transfected with the GFP vector was used as a control. The relative activity of firefly luciferase is shown after normalization with Renilla luciferase activity. The cell viability assay was conducted with a CellTiter-Glo Luminescent Cell Viability Assay kit by following the manufacturer’s instructions (Promega).

### 2.5. Immunofluorescence Assay (IFA)

For detection of HEV proteins in HepG2/C3A cells, IFA was carried out as previously reported [17] by using mouse anti-helicase monoclonal antibody (homemade), chimpanzee antibody against the HEV capsid protein (a gift from Suzanne Emerson at NIH), and rabbit anti-ORF3 product (a gift from Xiang-Jin Meng at Virginia Tech). The secondary antibodies used in IFA were DyLight 549 goat anti-mouse, anti-human, or anti-rabbit IgG (Rockland Immunochemicals, Inc., Gilbertsville, PA). The cell nucleus was stained with 4′6′-diamidino-2-phenylindole (DAPI) (Thermo Fisher). SlowFade Gold antifade reagent (Thermo Fisher) was used for coverglass mounting before confocal fluorescence microscopy.

### 2.6. Western Blot Analysis

Cells were lysed in Laemmli sample buffer. The lysate samples were subjected to sodium dodecyl sulfate-polyacrylamide gel electrophoresis (SDS-PAGE) and Western blotting, as previously described [17]. Primary antibodies against green fluorescence protein (GFP) (Santa Cruz Biotechnology, Inc., Dallas, TX), IRF3 and phosphorylated IRF3-S396 (Cell Signaling Technology, Danvers, MA), HA (Thermo Fisher), Myc (Thermo Fisher), FLAG (Sigma-Aldrich, St. Loius, MO), GAPDH (Santa Cruz), TBK1 and phosphorylated TBK1 (Cell Signaling), IRF3 (Santa Cruz), MAVS (Santa Cruz), and β-tubulin (Sigma) were used in the blotting. Horseradish peroxidase-conjugated secondary antibodies used in this study were goat anti-rabbit or goat anti-mouse IgG (Rockland Immunochemicals). The chemiluminescence signal was collected and analyzed digitally using a ChemiDoc XRS imaging system (Bio-Rad Laboratories, Hercules, CA) and the Quantity One Program, version 4.6 (Bio-Rad). 

### 2.7. Reverse Transcription and Quantitative PCR (RT-qPCR)

Total RNA was isolated from HepG2/C3A cells with TRIzol reagent (Thermo Fisher) and used for reverse transcription with avian myeloblastosis virus (AMV) reverse transcriptase, along with oligo(dT) and a random 15-mer oligo. Real-time PCR was performed with SYBR green supermix (Thermo Fisher) [22]. Transcripts of RPL32 (ribosomal protein L32) were also detected as the reference gene. Primers for IFN-β and RPL32 were described previously [23].

### 2.8. Co-Immunoprecipitation (Co-IP)

IP was conducted as described [17] with the antibodies against Myc, HA, HEV ORF2 (homemade) or FLAG. Protein G agarose (KPL Inc., Gaithersburg, MD) was used following the manufacturer’s instructions. The IP samples were subjected to immunoblotting analysis with the antibodies against Myc, HA, HEV ORF2, or FLAG. 

### 2.9. Statistical Analysis

Differences in indicators between treated samples, such as the IFN-β mRNA level between HEV-infected and mock-infected cells were assessed by the Student *t*-test. A two-tailed *p* value of less than 0.05 was considered significant.

## 3. Results

### 3.1. HEV Kernow-C1 Strain Inhibits Poly(I:C)-Induced IFN Production, and Only the Capsid Protein Is Detectable in Infected HepG2/C3A Cells

**Figure 1:**
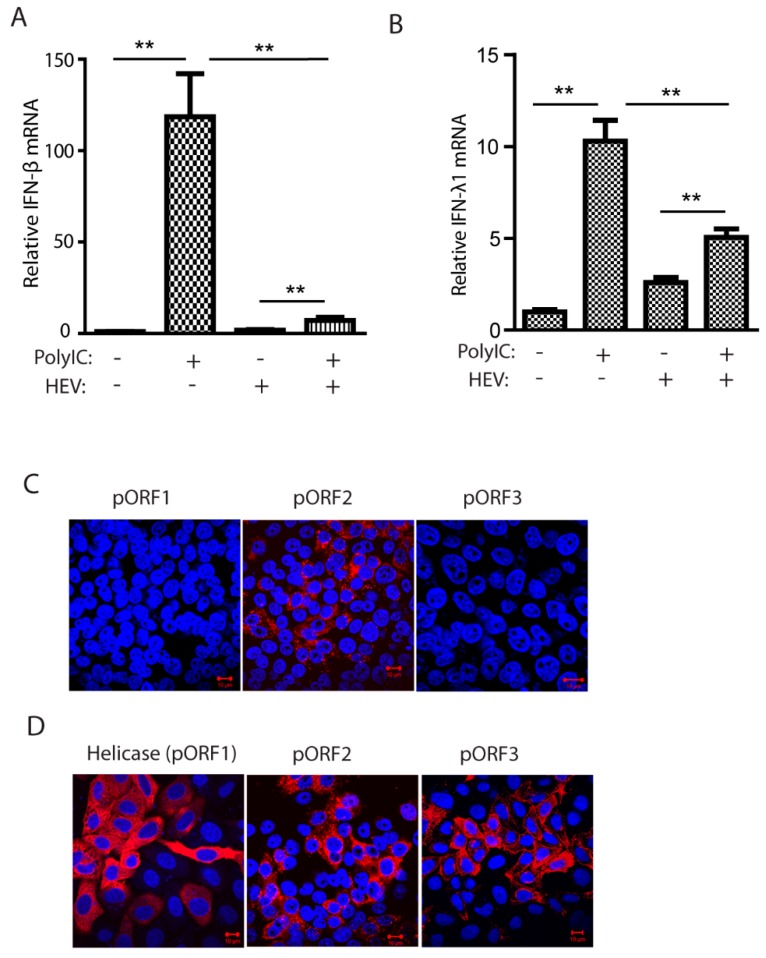
Hepatitis E virus (HEV) infection downregulates interferons (IFN) induction. (**A**) HEV inhibits poly(I:C)-induced IFN-β expression in HepG2/C3A cells. The cells were infected by HEV Kernow-P6 strain and transfected with poly(I:C) at 6 μg/mL and, 8 h later, harvested for RNA isolation and real-time PCR. Significant differences between the poly(I:C)-treated samples are denoted with ** for *p* < 0.01. (**B**) HEV inhibits poly(I:C)-induced IFN-λ1 expression in HepG2/C3A cells. (**C**) Indirect immunofluorescence assay (IFA) of the HepG2/C3A cells infected with Kernow-p6. Antibodies against HEV helicase (pORF1), pORF2, and pORF3 proteins were used. Red indicates HEV-positive staining, and blue is DAPI staining of nuclear DNA. The scale bar in the lower right of the images denotes 10 μm. (**D**) IFA of HeLa cells transiently transfected with plasmids of helicase, ORF2, and ORF3. IFA was done with antibodies against HEV helicase, pORF2, and pORF3 proteins. The scale bar in the lower right of the images denotes 10 μm.

To confirm that HEV Kernow-C1 strain impairs IFN production, we transfected HEV-infected HepG2/C3A cells with low molecular weight poly(I:C) at 6 μg/mL and determined IFN-β and IFN-λ1 mRNA levels for both type I and III interferons, respectively. Compared to mock-infected HepG2/C3A cells, both the IFN-β and IFN-λ1 mRNA levels in the HEV-infected cells were significantly lower (Figure 1 A,B). However, the inhibition of type III IFN appears less than inhibition of type I IFN. The IFA results showed that in the Kernow-infected HepG2/C3A cells, only the capsid protein could be detectable, while ORF1 and ORF3 products were below the detection level (Figure 1C), indicating the abundance of the capsid protein. To verify the antibodies against the individual viral proteins, HEV helicase, ORF2, and ORF3 were transiently expressed in HeLa cells. IFA results showed that all the antibodies were efficient in the detection of the target proteins (Figure 1D), suggesting that the expression of ORF1 and ORF3 products in the Kernow-infected cells is low. 

### 3.2. The Capsid Proteins of Both Genotype 1 and 3 HEV Strains Impair Poly(I:C)-Induced IFN Production 

**Figure 2:**
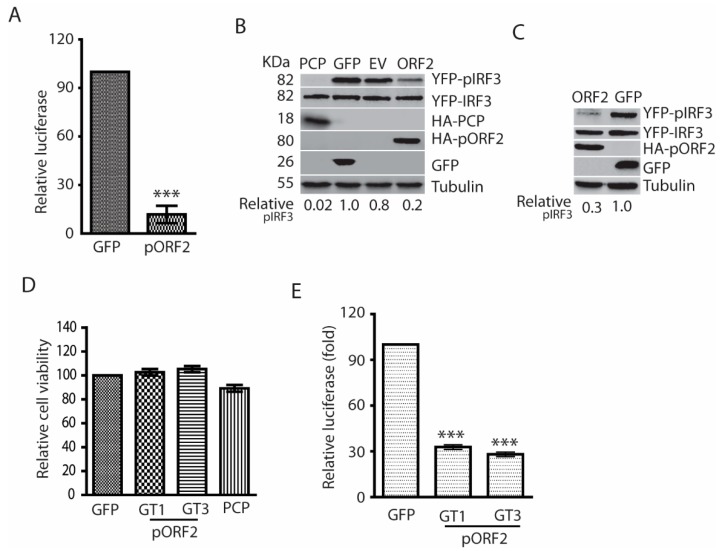
The capsid proteins of both genotype 1 and 3 HEV inhibit poly(I:C)-induced IFN production. (**A**) The capsid protein of Kernow (genotype 3, GT3) inhibits IFN-β reporter expression in HEK293T cells. The cells were transfected with plasmids of GT3 ORF2 and IFN-β reporter. At 36 h post-transfection (hpt), the cells were transfected with poly(I:C) at 1 μg/mL. The luciferase activity was detected after 24-h treatment. “***” denotes *p* < 0.001. (**B**) The capsid protein of HEV Kernow inhibits poly(I:C)-induced IRF3 phosphorylation. The 293T cells were co-transfected with plasmids of Kernow ORF2 and YFP-IRF3. Poly(I:C) treatment was conducted 24 h later. The cells were harvested for Western blotting (WB) after 8-h treatment. The PCP of HEV ORF1, GFP and empty vector (EV) were included as controls. WB with antibodies against phosphor-IRF3 (pIRF3), IRF3, HA tag, ORF2 product (pORF2), GFP and tubulin was done. The relative level of pIRF3 after normalization with tubulin is shown below the images. The molecular weights of the proteins are indicated on the left. (**C**) HEV Sar55 (genotype 1, GT1) ORF2 blocks IRF3 phosphorylation induced by poly(I:C). The GT1 ORF2 plasmid and YFP-IRF3 were co-transfected to HEK293T for pIRF3 test. Transfection and WB were conducted as described in “B” above. (**D**) Relative cell viability of HEK293T cells after transfection. The cells were transfected with plasmids of GFP, HEV ORF2, and PCP for 48 h before the cell viability assay. (E). HEV pORF2 downregulates Sendai virus-induced IFN-β expression in HEK293T cells. The cells were transfected with plasmids of HEV ORF2 (GT1 and GT3) and IFN-β reporter. At 24 hpt, the cells were infected with Sendai virus at an MOI of 5. Luciferase assay was done 24 h post-infection. “***” denotes *p* < 0.001.

The X and PCP domains of genotype 1 HEV are known to inhibit IFN production [17]. However, the expression of the nonstructural proteins in HEV-infected patients, animal models, or cultured cells is generally at low abundance [10,11], which suggests their roles in antagonizing IFN induction might be limited. We speculated that the capsid protein, the most abundant among all the viral proteins during HEV infection, might play a role in downregulating IFN signaling to generate a conducive environment for HEV replication. To test this speculation, HEK293T cells were transfected with the ORF2 plasmid of HEV Kernow-C1 and IFN-β luciferase reporter, followed by poly(I:C) stimulation on the following day. The luciferase reporter assay results showed that the capsid protein could significantly inhibit the poly(I:C)-induced IFN-β reporter expression to 12% compared with the GFP control (Figure 2A). Upon seeing the inhibition of IFN-β reporter expression, we wondered if the capsid protein has any effect on IRF3 as it is the main transcription activator for IFN-β expression. Indeed, the capsid protein inhibited the phosphorylation of IRF3 (pIRF3), whereas the total IRF3 protein level had minimal change in comparison with the empty vector control (Figure 2B). 

To exclude the possibility that the IFN inhibition is the sole feature of the capsid protein of Kernow-C1, a genotype 3 strain, we transfected HEK293T cells with the ORF2 plasmid of Sar55, a genotype 1 strain. Similarly, the Sar55 ORF2 product also blocked poly(I:C)-stimulated IRF3 phosphorylation (Figure 2C). To assess the possible cytotoxic effect of the transfection to the cells, cell viability assay was conducted. The result showed that the overexpression of the capsid proteins had minimal effect on the cell viability compared with GFP control (Figure 2D). 

In the experiments above, we used poly(I:C), a synthetic analog of dsRNA, to stimulate the cells. To mimic natural viral infection, the HEK293T cells were infected with Sendai virus for 24 h in the presence of the capsid protein expression. Compared with the GFP control, both the capsid proteins of genotype 1 and 3 HEV significantly inhibited the expression of IFN-β reporter (Figure 2E). These results demonstrated that the HEV capsid protein could inhibit IFN induction by blocking the phosphorylation of IRF3. 

### 3.3. The Capsid Protein Inhibits TBK1-Mediated Phosphorylation of IRF3

**Figure 3:**
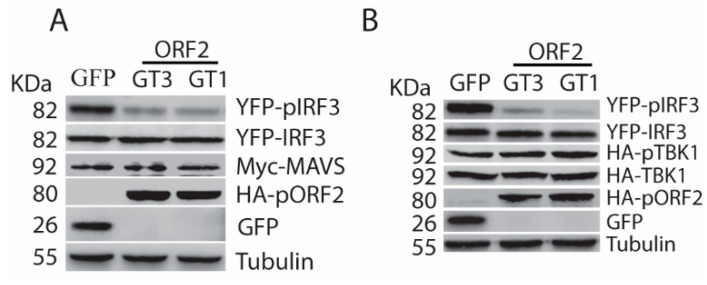
The capsid protein inhibits IFN induction by blocking TBK1-mediated IRF3 phosphorylation. (**A**) The capsid protein of HEV GT1 and GT3 blocks MAVS-induced IRF3 activation. The HEK293T cells were co-transfected with ORF2, IRF3, and MAVS plasmids. The cells were harvested at 24 h later for WB. The molecular weights of the proteins are indicated on the left. (**B**) The capsid protein blocks TBK1-induced IRF3 activation. The HEK293T cells were co-transfected with ORF2, IRF3, and TBK1. Transfection and WB were conducted as described in “A” above.

Overexpression of some individual upstream molecules of the RLR pathway, such as RIG-I, MAVS, and TBK1, is sufficient to activate the signaling pathway [17]. To determine the mechanism of the capsid protein-mediated inhibition of IFN signaling, we transfected HEK293T cells with the plasmids of ORF2 and MAVS, or ORF2, and TBK1. Western blotting showed that pORF2 of both HEV genotypes could inhibit the MAVS-induced phosphorylation of IRF3, whereas they had minimal effect on MAVS and IRF3 protein levels (Figure 3A). Similarly, the capsid proteins also downregulated TBK1-induced IRF3 phosphorylation (Figure 3B). However, phospho-TBK1 (pTBK1) level had minimal change in the presence of HEV ORF2 expression, suggesting that the capsid protein does not affect the TBK1 activation. These results indicate that the capsid protein targets the TBK1 function to block the IRF3 activation. 

### 3.4. The Capsid Protein Interacts with TBK1 to Inhibit the IRF3 Activation

**Figure 4:**
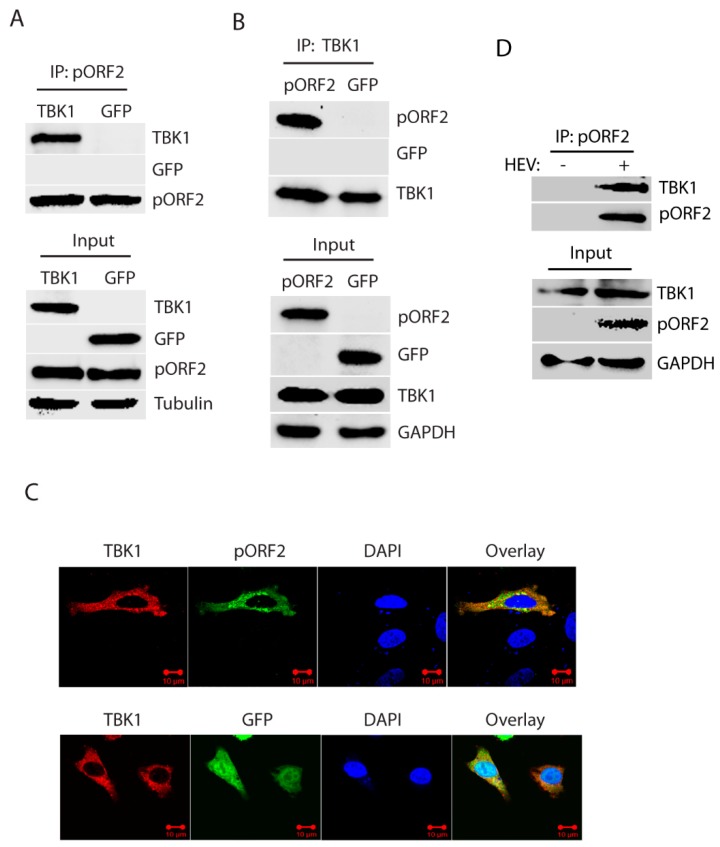
The capsid protein interacts with TBK1. (**A**) TBK1 presents in the Co-IP complex of pORF2. HEK293-IRF3 stable cells were transfected with pORF2 and TBK1 plasmids. Co-IP with pORF2 antibody was done at 36 hpt, followed by WB with antibodies against TBK1, GFP, and pORF2. WB of input cell lysate is shown in the lower panel. (**B**) pORF2 presents in the Co-IP complex of TBK1 from HEK293-IRF3 stable cells. HEK293-IRF3 stable cells were transfected with pORF2 and TBK1 plasmids. Co-IP with FLAG-TBK1 was done at 36 hpt, followed by WB with antibodies against pORF2, GFP, and TBK1. (**C**) The capsid protein co-localizes with TBK1. HeLa cells were transfected with pORF2 and TBK1 plasmids. IFA and confocal microscopy were done at 36 hpt. Green stands for ORF2, red indicates TBK1, and blue is DAPI staining. The scale bar in the lower right of the images denotes 10 µm. (**D**) IP of pORF2 co-precipitates TBK1 in HEV-infected HepG2/C3A cells. The cells were infected with HEV Kernow-C1 strain. Mock-infected cells were included as a control.

Upon activation, TBK1 is recruited to MAVS to induce phosphorylation of MAVS, followed by the recruitment of IRF3 to MAVS and subsequent phosphorylation of IRF3 [16,24]. The activated IRF3 is then dimerized, dissociated from MAVS, and translocated into the nucleus. To investigate whether pORF2 protein impairs IRF3 phosphorylation through interaction with TBK1, we transfected the HEK293-IRF3 stable cell line that was established earlier in the lab [17]. Transfection of the cells with ORF2 and TBK1 plasmids was conducted, followed by co-IP. The results showed that the IP of pORF2 co-precipitated TBK1 (Figure 4A). Similarly, IP of TBK1 co-precipitated pORF2 (Figure 4B). GFP was included as a control and did not co-precipitate with either pORF2 or TBK1. Western blotting of the whole cell lysate confirmed the expression of these proteins. To confirm the observation, HeLa cells were co-transfected with ORF2 and TBK1 plasmids, followed by IFA and confocal microscopy. The result showed that pORF2 had co-localization with TBK1, whereas GFP did not co-localize with the latter (Figure 4C). 

To further confirm the interaction of pORF2 with TBK1, we conducted co-IP using HEV Kernow-infected HepG2/C3A cells. The results showed that pORF2 co-IP precipitated TBK1 in the HEV-infected cells, whereas the antibody against pORF2 did not precipitate TBK1 in the mock-infected cells (Figure 4D). These results suggest that pORF2 interacts with TBK1 to block IRF3 phosphorylation.

### 3.5. The Capsid Protein Blocks the Phosphorylation and Dissociation of IRF3 from MAVS

**Figure 5:**
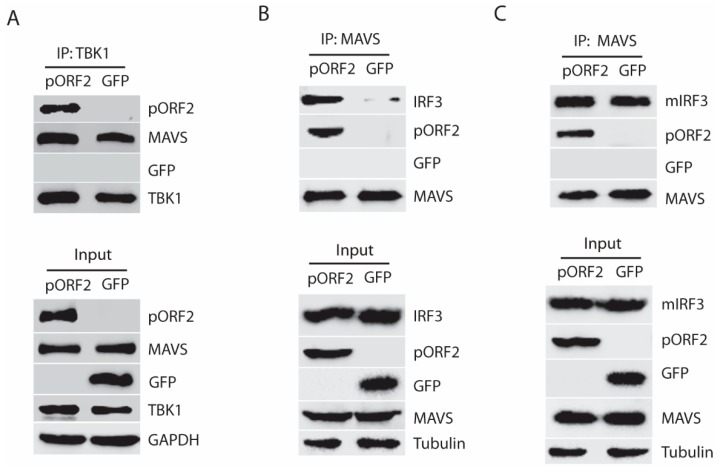
The capsid protein inhibits the dissociation of IRF3 from the MAVS-TBK1 complex. (**A**) TBK1 interacts with ORF2 in the presence of MAVS. HEK293T cells were co-transfected with TBK1, pORF2, and MAVS, and the Co-IP was performed at 36 hpt with the HA-TBK1 antibody. The IP product and input were subjected to WB detection with pORF2, TBK1, MAVS, and GFP antibodies. (**B**) MAVS IP precipitates IRF3 and pORF2, whereas much less IRF3 in the control of GFP. The HEK293T cells were transfected with plasmids of Myc-MAVS, IRF3, and pORF2. Co-IP was done with the Myc-MAVS antibody, and the product was detected by WB with antibodies against MAVS, IRF3, and pORF2. WB of input cell lysate is shown in the lower panel. (**C**) The presence of ORF2 does not affect mutant IRF3 interaction with MAVS. The HEK293T cells were transfected with plasmids of Myc-MAVS, mutant IRF3 (S385A/S386A), and pORF2. The Co-IP detection was done as panel B.

Given the fact that pORF2 protein interacts with TBK1 without affecting its phosphorylation level, we speculated that the binding of TBK1 and pORF2 protein might interfere with the interaction between TBK1 and MAVS, or between MAVS and IRF3. To test this, co-IP of TBK1 was done in the presence of pORF2 or GFP. Western blotting of the TBK1 precipitates showed the presence of MAVS with or without pORF2 (Figure 5A), which indicates that pORF2 does not affect the interaction of TBK1 and MAVS. Next, we conducted co-IP of MAVS to test if the interaction of MAVS and IRF3 was affected by pORF2. Interestingly, the MAVS IP in the presence of pORF2 co-precipitated more IRF3 than that of GFP control (Figure 5B). The MAVS IP also co-precipitated pORF2 but not GFP. Since IRF3 is expected to dissociate from MAVS after being phosphorylated and homodimerized, the higher level of IRF3 in the MAVS/pORF2 precipitates suggests that pORF2 might affect the dissociation of IRF3 from MAVS.

To test this, we constructed a mutant IRF3 (S385A/S386A), which cannot be phosphorylated and dissociated from MAVS [16]. Therefore, the interaction of MAVS and the mutant IRF3 (mIRF3) in the presence of pORF2 or GFP should be similar. Indeed, co-IP of MAVS from cells with pORF2 expression co-precipitated the mIRF3 to a level similar to the co-IP of MAVS from the cells with GFP expression (Figure 5C), suggesting that pORF2 does not block the recruitment of IRF3 to the complex. These results indicate that the pORF2 binds with MAVS-TBK1-IRF3 complex and blocks the phosphorylation of IRF3 and its subsequent dissociation from MAVS. 

### 3.6. The First 111 Residues of the Capsid Protein Are Responsible for the Inhibition of TBK1-Induced IRF3 Phosphorylation

**Figure 6:**
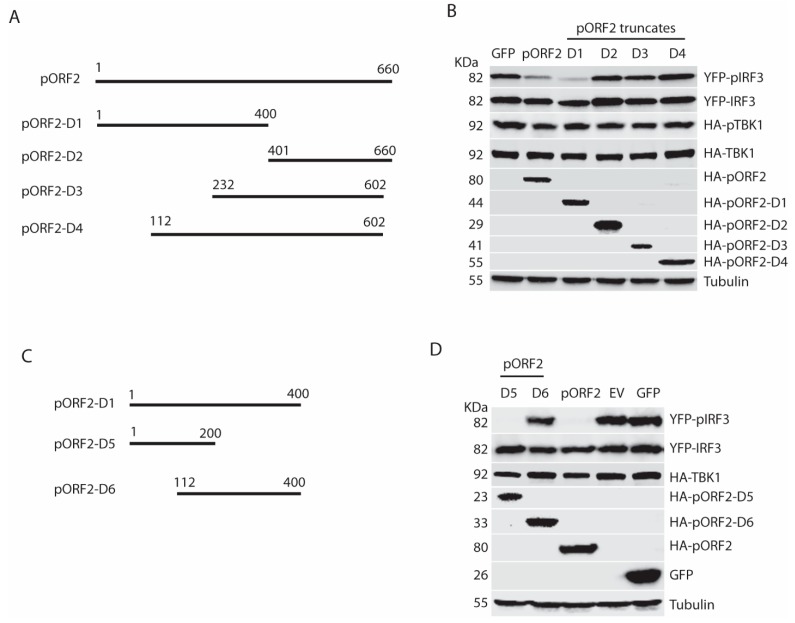
The first 111 aa of the capsid protein is responsible for the inhibition of IRF3 activation. (**A**) Schematic illustration of ORF2 truncation mutants. (**B**) The pORF2-D1 inhibits TBK1-mediated IRF3 activation. The HEK293-IRF3 stable cell line was co-transfected with ORF2 truncations and TBK1 plasmids, and at 36 hpt, the samples were subjected to WB for pIRF3 detection. The molecular weights of the proteins are indicated on the left. (**C**) Schematic illustration of ORF2 truncation mutants D5 and D6. (**D**) The pORF2-D5 inhibits IRF3 activation. Plasmids of ORF2 D5 and D6 were co-transfected to HEK293-IRF3 cells, and at 36 hpt, the samples were subjected to WB for pIRF3 detection.

To determine the functional domain of pORF2 in the inhibition of IFN signaling, we constructed four pORF2 truncates based on the predicted possibility of the surface orientation of the pORF2 polypeptide (Figure 6A). These four ORF2 truncates were tested for their effects on TBK1-induced IRF3 phosphorylation in HEK293-IRF3 stable cells. Western blotting results showed that pORF2-D1 inhibited TBK1-induced IRF3 phosphorylation, whereas the other three truncates had a minimal effect (Figure 6B). The pORF2-D1 expression had no detectable effect on the TBK1 level or its phosphorylation. The difference between pORF2-D1 and the other three truncates is that pORF2-D1 contains the first 111 aa residues.

To further confirm this observation, two additional truncates, pORF2-D5 (spans 1-200aa) and pORF2-D6 (112-400aa), were prepared based on pORF2-D1 (Figure 6C). Transient transfection of HEK293-IRF3 stable cells with pORF2-D5, pORF2-D6, and TBK1 plasmids was done. Compared with the empty vector or GFP controls, pORF2-D5 blocked IRF3 phosphorylation to a level similar to that of full-length pORF2, while pORF2-D6 had much less effect (Figure 6D). These results demonstrated that the first 111 residues of the capsid protein were essential for the inhibition of IRF3 phosphorylation.

### 3.7. The Arginine-Rich-Motif (ARM) within the N Terminus of the Capsid Protein Is Indispensable for Blocking IRF3 Phosphorylation

**Figure 7:**
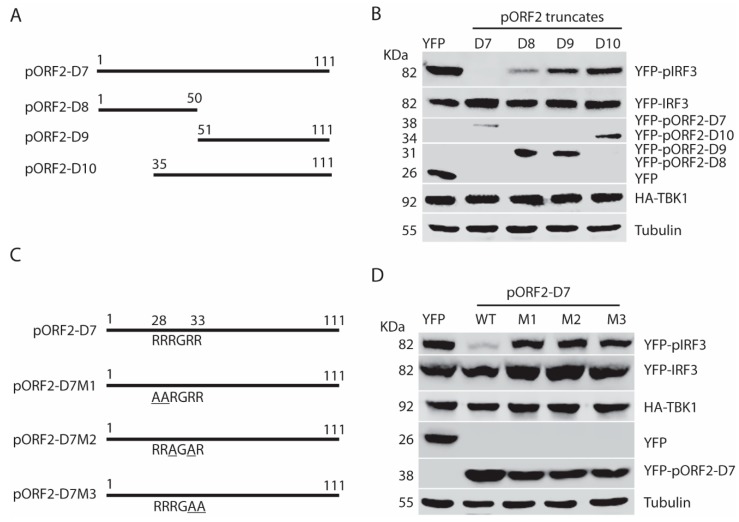
Arginine-rich motif aa28-33 (RRRGRR) in the first 111 aa of the capsid protein is indispensable for inhibition of IRF3 phosphorylation. (**A**) Schematic illustration of truncation mutants of the first 111 aa of the capsid protein. (**B**) Domain screening of ORF2 truncations in the inhibition of TBK1-mediated IFN induction. The HEK293-IRF3 stable cell line was co-transfected with ORF2 N-terminal truncations and TBK1 plasmids for 36 h, and the samples were harvested for WB. (**C**) Schematic illustration of mutants of arginine-rich motif aa28-33 in the capsid protein. (**D**) All three mutants lose the inhibition of IRF3 activation. The HEK293-IRF3 stable cell line was transfected with three mutant plasmids and TBK1. The pIRF3 level was detected at 36 hpt by WB.

To further map the functional domain of the first 111aa of pORF2, pORF2-D7, pORF2-D8, pORF2-D9, and pORF2-D10 were constructed (Figure 7A). These truncated constructs were co-transfected with TBK1 plasmid into HEK293-IRF3 cells. Western blotting results showed pORF2-D7 and pORF2-D8 blocked TBK1-induced activation of IRF3 in the cells, whereas the rest two truncates had much less effect (Figure 7B).

From sequence analysis, we noticed a conserved ARM that is located at aa 28 to 33 of the capsid protein of both genotype 1 and 3 HEV. One of the main functions of the ARM is RNA binding and processing, and many viral ARMs are responsible for the packaging of viral RNA [25,26,27,28]. In addition to the RNA-binding activity, the ARM also participates in protein-protein interaction [29]. To determine whether the ARM in the capsid protein has any role in the inhibition of IRF3 activation, site-directed mutagenesis of pORF2-D7 constructs was conducted, and three mutant plasmids were generated with each having two arginines changed to alanines (Figure 7C). These plasmids of mutant and wild-type pORF2-D7 were transfected into HEK293-IRF3 stable cells along with TBK1. Western blotting results showed that all three mutants failed to inhibit TBK1-induced activation of IRF3 (Figure 7D). These results indicate that the ARM in the first 111 aa of pORF2 is crucial for the inhibition of IRF3 phosphorylation.

**Figure 8:**
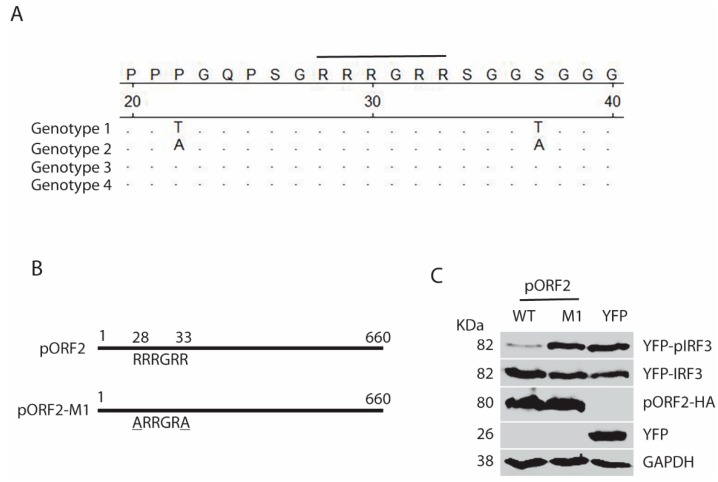
The arginine-rich motif aa28-33 (RRRGRR) in the full-length capsid protein is indispensable for the inhibition of IRF3 phosphorylation. (**A**) The arginine-rich motif aa28-33 (RRRGRR) is conserved across the four genotypes of HEV that infect humans (GenBank accession numbers for the genotype 1 to 4 strains in the alignment are: LC061267.1, KX578717.1, AF444003.1, and AB220979.1, respectively). The top amino acid sequence denotes the consensus of the four genotypes. The line above the residues denotes the arginine-rich motif aa28-33 (RRRGRR). The residues that are identical to the consensus sequence are denoted by “.” in the alignment. The numbers below the consensus sequence indicate the position in the full-length pORF2. (**B**) Schematic illustration of the full-length pORF2 and the point mutations in the arginine-rich motif: R28A and R33A. The alanine residues in the mutation sites of pORF2-M1 are underlined. (**C**) Mutant pORF2 protein loses the inhibition of IRF3 activation. HEK293-IRF3 cells were transfected with wild-type (WT) pORF2, pORF2-M1, or YFP plasmids. The cells were treated with poly(I:C) for 8 h before harvested for WB.

To further confirm the role of the ARM in the full-length pORF2, we conducted sequence analysis of genotype 1 to 4 that are known to infect humans. The ARM is conserved with identical residues across the strains of the four genotypes in the sequence analysis (Figure 8A). To determine if the ARM is also functional in the full-length pORF2, we conducted site-directed mutagenesis and generated a mutant pORF2-M1 with R28A and R33A mutations (Figure 8B). As expected, the pORF2-M1 had minimal inhibition of poly(I:C)-induced IRF3 phosphorylation while the wild-type pORF2 inhibited it (Figure 8C). Notably, pORF2 with HA tag at the C-terminus blocked poly(I:C)-induced IRF3 phosphorylation (Figure 8C), similarly to the pORF2 with HA tag at the N-terminus (Figure 2 and Figure 3). These results demonstrate that pORF2 inhibits the IFN induction via the ARM-mediated inhibition of IRF3 phosphorylation.

## 4. Discussion

**Figure 9:**
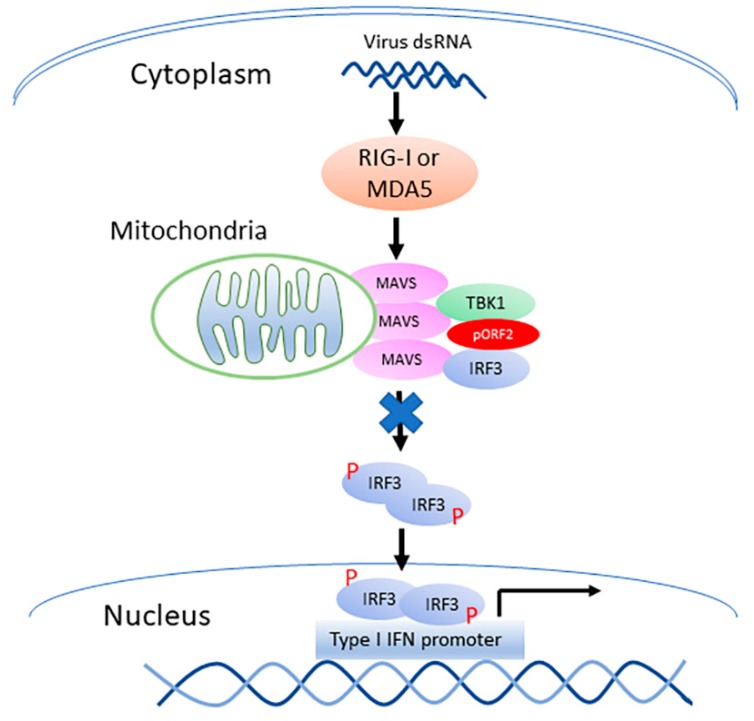
Model for the capsid protein inhibition of TBK1-mediated IRF3 phosphorylation. “P” denotes phosphorylation of IRF3. The capsid protein is denoted by “pORF2”. “X” on the arrow from MAVS to IRF3 indicates blocking the phosphorylation of IRF3.

HEV infection elevates the RIG-I and MDA5 protein levels in hepatoma cells, and causes the persistent production of type III IFN but not type I IFN [12]. Unlike the HAV and HCV that cleaves MAVS, HEV does not do so to antagonize the IFN induction [12]. HEV ORF1 products, X and PCP, are capable of inhibiting the induction of type I IFNs [17]. However, the ORF1 products in the HEV-infected cells may be at low abundance and hard to be detected in IFA with the current antibodies [12]. The capsid protein is the most abundant among all HEV proteins. In this study, we demonstrate that the capsid protein inhibits the production of both type I and type III IFNs. Notably, HEV significantly inhibits the poly(I:C) induction of type III IFN, although Kernow infection induces a persistent induction of type III IFN at low and tolerable level [12]. Our results showed that the capsid protein exerted this inhibition by interacting with the MAVS-TBK1-IRF3 complex to block the phosphorylation of IRF3 (Figure 9). Our data also demonstrated that the ARM in the N-terminus of the capsid protein was indispensable for the inhibition of IRF3 activation.

In chronically infected patients, the ORF2 antigen level in the serum is significantly higher than that in the acute cases [8]. Also, in patients with chronic infection, the capsid antigen of genotype 3 HEV can still be detected for more than 100 days after virus RNA elimination by ribavirin application, which suggests an essential role of ORF2 protein in the disease progression [8].

After the HEV infection, the virus must employ effective strategies to antagonize the innate immunity to survive in the host cells. ORF2 product interacts with F-box protein β-TRCP to decrease the ubiquitination of IκBα, and subsequently inhibit the activity of NF-κB [30]. In addition, ORF2 product activates the promoter of an anti-apoptotic protein, Chop, without inducing cell apoptosis [31]. Our data demonstrate that the capsid protein of HEV can downregulate the IFN production via its ARM in the N-terminal domain, which reveals another strategy of HEV in evading host innate immune response.

In the IFN induction pathway, the MAVS-TBK1-IRF3 complex has consistently been a focusing topic, and the accurate dynamic biologic process of the complex formation remains incompletely understood. In the resting state, TBK1 is sequestrated and silenced in the cytoplasm by protein phosphatase 1A (PPM1A) [32]. TIR domain-containing adapter molecules (TRAFs) are demonstrated to pre-associate with TBK1 [33]. Upon RLR activation, MAVS recruits TRAFs and subsequently activates TBK1. When MAVS is expressed at a high level, it can dissociate the TBK1/PPM1A complex to antagonize PPM1A-mediated inhibition. Four adaptor proteins, including TANK (TRAF family member-associated NF-kB activator), NAP1 (NF-kB activating kinase-associated protein 1 or 5-azacytidine-induced protein 2 (Azi2)), SINTBAD (similar to NAP1 TBK1 adaptor), and optineurin, are known to directly interact with TBK1 at the C-terminal domain via a shared TBK1 binding domain [34,35]. However, the binding of NAP1 and TBK1 is irrespective of the dimerization and phosphorylation of TBK1 [36]. TANK is demonstrated to facilitate the activation of IFN signaling through association with a series of proteins, such as TBK1, NF-κB essential modulator (NEMO), TRAF1, and TRAF3 [37]. Thus, the IFN induction signaling through the MAVS complex involves the participation of complicated components and a dynamic process. During virus infection, the viruses could interact with host proteins to dampen the functional assembly of the MAVS complex [38]. Our data shows that the HEV capsid protein interacts with the MAVS-TBK1-IRF3 complex, which results in the blocking of the IRF3 phosphorylation and dissociation from the complex. This conclusion is based on the co-IP results of multiple experiments. The pORF2 interaction with the TBK1-MAVS-IRF3 complex was confirmed with co-transfection of pORF2 and mutant IRF3 that cannot be phosphorylated and dissociated from MAVS. The similar level of mutant IRF3 in co-IP in the presence of pORF2 or GFP suggests that pORF2 did not interfere with the MAVS recruitment of IRF3. However, the exact mechanism of pORF2 interaction with the complex needs a structural study to reveal, which is beyond the scope of this study.

By sequence analysis, an ARM is found within the N-terminal domain. The ARM is conserved across the four genotypes that are known to infect humans. The ARM ubiquitously interacts with RNA and is involved in transcriptional regulation and cell proliferation [39]. Besides, the ARM also participates in protein-protein interactions. For instance, the ARM of ring finger protein 4 (RNF4), a ubiquitin E3 ligase, acts as a recognition motif to recruit target selectively and promote the degradation of Kruppel-associated box domain-associated protein 1 (KAP1) [29]. In this study, we demonstrated that the N terminal domain of the HEV capsid protein inhibited the TBK1-induced activation of IRF3, while the mutation of the ARM within the N terminus abolished this effect. This indicates an essential role of this motif in the IFN antagonization by pORF2. All the arginine residues in the ARM are essential for the pORF2 inhibition of IRF3 phosphorylation, as a mutation in them abolished the function. The essential role of the ARM was also confirmed in the full-length pORF2 as the mutation of the first, and the last arginine abolished the poly(I:C)-induced IRF3 phosphorylation. Our data suggest that the intact ARM is indispensable in the pORF2 interaction with the TBK1-MAVS-IRF3 complex to inhibit the IRF3 phosphorylation.

In conclusion, the HEV capsid protein inhibits IFN production via interaction with the MAVS-TBK1-IRF3 complex and consequent blocking of IRF3 phosphorylation. The ARM in the N-terminus of the capsid protein is indispensable to the inhibition. This study provides further insight into the HEV evasion of host innate immunity.

## Figures and Tables

**Table 1:** Primers used in this study.

Primer ^a^	Sequence (5′ to 3′) ^b^	Target
ORF2-T1F	CCG*CTCGAG*GTCGCTCCGGCCCATGACA	T1 ORF2
ORF2-T1R	G*GCGGCCGC*TTACTATAACTCCCGAGTTTTAC	T1 ORF2
ORF2-T3F	G*CTCGAG*TGCCCTAGGGTTGTTCTGCTGCTGTTC	T3 ORF2
ORF2-T3R	G*GCGGCCGC*TTAAGACTCCCGGGTTTTGCCTACCTCCG	T3 ORF2
ORF2-D1F	ATCTGCTC*GAATTC*GCCACCATGTGCCCTAGGGTTGTTC	ORF2 D1
ORF2-D1R	GC*CTCGAG*AGGGCGGGAGTAGAACA	ORF2 D1
ORF2-D2F	C*CTCGAG*GTTGTCTCGGCCAATGGCGA	ORF2 D2
ORF2-D2R	A*GCGGCCGC*TTAAGACTCCCGGGTTTTGCC	ORF2 D2
ORF2-D3F	T*CTCGAG*GTTAGGATTTTGGTCCAGCC	ORF2 D3
ORF2-D3R	A*GCGGCCGC*TTAGGCTAATACACCCACCGC	ORF2 D3
ORF2-D4F	T*CTCGAG*GCTGTATCACCAGCCCCTGA	ORF2 D4
ORF2-D4R	A*GCGGCCGC*TTAGCTAATACACCCACCGCGGAGA	ORF2 D4
ORF2-D7-R	AC*CTCGAG*AGTCAACGGCGCAGCCCCAG	ORF2 D7
ORF2-D8-R	A*CTCGAG*GGGCTGAGAATCAACCCTGT	ORF2 D8
ORF2-D9-F	T*GAATTC*GCCACCATGTTCGCCCTCCCCTATATTCA	ORF2 D9
ORF2-D10-F	A*GAATTC*GCCACCATGGGCGGTGCCGGCGGTGGTTTC	ORF2 D10
mIRF3-F1	C*GAATTC*GGAACCCCAAAGCCACGGATCC	Mutant IRF3
mIRF3-R1	CTCCAGGGCGGCGGCACCCCCTACCCGGGCCATTTCTA	Mutant IRF3
mIRF3-F2	GGTAGGGGGTGCCGCCGCCCTGGAGAATACTGTGGACCTGC	Mutant IRF3
mIRF3-R2	A*CTCGAG*TCAGCTCTCCCCAGGGCCCT	Mutant IRF3
TBK1-F	AACGTCTC*GAATTC*CAGAGCACTTCTAATCATCTG	TBK1
TBK1-R	C*CTCGAG*CTAAAGACAGTCAACGTTGCGAAG	TBK1
ORF2-N-M1F	CAGCCGTCTGGCGCTGCTCGTGGGCGGCGCAGCGGCGGTGC	ORF2-D7-M1
ORF2-N-M1R	CTGCGCCGCCCACGAGCAGCGCCAGACGGCTGGCCGGCCGGT	ORF2-D7-M1
ORF2-N-M2F	GTCTGGCCGTCGTGCTGGGGCTCGCAGCGGCGGTGCCGGCGGT	ORF2-D7-M2
ORF2-N-M2R	ACCGCCGCTGCGAGCCCCAGCACGACGGCCAGACGGCTGGCCG	ORF2-D7-M2
ORF2-N-M3F	TGGCCGTCGTCGTGGGGCCGCCAGCGGCGGTGCCGGCGGTGGT	ORF2-D7-M3
ORF2-M1-F1	CTGCCCGCGCCACCGGCCGGCCAGCCGTCTGGCGCTCGTCGTGGGC GGGCCAGCGGCGGTGC	ORF2-M1
ORF2-M1-F3	ACCGTCTCGAATTCGCCACCATGTGCCCTAGGGTTGTTCTGCTGCT GTTCTTCGTGTTTCTGCCTATGCTGCCCGCGCCACCGGCCGGCCAG	ORF2-M1
KORF2-R5	A*CTCGAG*AGACTCCCGGGTTTTGCCTACCTCCGTT	ORF2
ORF2-N-M3R	CCGGCACCGCCGCTGGCGGCCCCACGACGACGGCCAGACGGC	ORF2-D7-M3

^a^ F: forward primer, R: reverse primer. The “m” before a primer name indicates the primer is designed for point mutation. All primers of ORF2 are based on the sequence of HEV Kernow-C1 P6 sequence (GenBank accession# JQ679013) except indicated above. ^b^ The italicized alphabets in primer sequence indicate restriction enzyme cleavage sites for cloning into target vectors.
